# Tumor Cell Survival Factors and Angiogenesis in Chronic Lymphocytic Leukemia: How Hot Is the Link?

**DOI:** 10.3390/cancers17010072

**Published:** 2024-12-29

**Authors:** Marianne Ayoub, Santos A. Susin, Brigitte Bauvois

**Affiliations:** Centre de Recherche des Cordeliers, Sorbonne Université, Université Paris Cité, Inserm UMRS 1138, Drug Resistance in Hematological Malignancies Team, F-75006 Paris, France; marianneayoub99@gmail.com (M.A.); santos.susin@sorbonne-universite.fr (S.A.S.)

**Keywords:** angiogenesis, angiopoietin-2, chemokine, drug resistance, leukemia, migration, matrix metalloproteinase-9, signaling, survival, vascular endothelial growth factor

## Abstract

Chronic lymphocytic leukemia (CLL) is characterized by the accumulation of neoplastic CD5^+^/CD19^+^ B lymphocytes in the blood. These cells migrate to and proliferate in the bone marrow and lymphoid tissues. Despite the development of new therapies for CLL, drug resistance and disease relapse still occur; novel treatment approaches are therefore still needed. Inhibition of the angiogenesis involved in the progression of CLL might be a relevant therapeutic strategy. The literature data indicate that vascular endothelial growth factor, angiopoietin-2, and matrix metalloproteinase-9 are pro-angiogenic factors in CLL. A number of other CLL factors might have pro-angiogenic activity: fibroblast growth factor-2, certain chemokines (such as CXCL-12 and CXCL-2), tumor necrosis factor-α, insulin-like growth factor-1, neutrophil gelatinase-associated lipocalin, and progranulin. All these molecules contribute to the survival, proliferation, and migration of CLL cells. Here, we review the literature on these factors’ respective expression profiles and roles in CLL. We also summarize the main results of preclinical and clinical trials of novel agents targeting most of these molecules in a CLL setting. Through the eradication of leukemic cells and the inhibition of angiogenesis, these therapeutic approaches might alter the course of CLL.

## Simple Summary

In chronic lymphocytic leukemia (CLL), abnormal B lymphocytes accumulate in the bone marrow (BM) and secondary lymphoid tissues. The BM and lymph nodes support angiogenesis and increased vascularization. Although certain drugs approved by the US Food and Drug Administration improve clinical outcomes, some patients do not respond and others relapse. Interactions between CLL cells and the tissue microenvironment favor leukemic cell trafficking, survival, and proliferation via the production of soluble factors. Some of these factors exhibit pro-angiogenic properties. This review summarizes the biology of these molecules with survival/pro-angiogenic value, and provides a summary of new, selective inhibitors targeting these molecules (and their receptors) currently under evaluation in preclinical and clinical studies.

## 1. Introduction

Angiogenesis (i.e., the formation of new blood vessels from existing ones) is a complex process that encompasses extracellular matrix remodeling, the activation, migration and proliferation of endothelial cells (ECs), capillary differentiation, and anastomosis [1,2,3]. This process requires a number of interactions between a variety of cells and is controlled by soluble pro- and anti-angiogenic factors [1,2,3]. Dysregulation of angiogenesis is one of the hallmarks of cancer [1,2,3]. Tumor angiogenesis can be triggered by pro-angiogenic factors expressed by tumor cells, immune cells (including mast cells, natural killer cells, and dendritic cells) or stromal cells (including ECs, fibroblasts, macrophages and mesenchymental stem/stromal cells (MSCs)) in the tumor microenvironment [1,2,3,4,5]. The cytokines, chemokines, and growth factors that promote angiogenesis include vascular endothelial growth factor (VEGF), fibroblast growth factor-2 (FGF-2, also known as basic FGF), angiopoietin-2 (Ang-2), chemokines (CXC chemokine ligand (CXCL)-12 and CXCL-2), matrix metalloproteinase-9 (MMP-9), tumor necrosis factor-α (TNF-α), insulin-like growth factor-1 (IGF-1), neutrophil gelatinase associated lipocalin (NGAL), progranulin, and angiogenin [6,7,8,9,10,11,12,13,14,15,16,17,18,19,20,21,22]. Anti-angiogenic factors have also been identified and include endostatin, adiponectin, angiostatin, and thrombospondin (TSP-1) [11,23].

Pro-angiogenic factors can stimulate angiogenesis directly or indirectly. Firstly, they may act directly by binding to the cognate receptor on ECs and thus induce cell proliferation and/or migration (as is the case for VEGF, FGF-2, CXCL-12, angiogenin, IGF-1, progranulin, MMP-9, and Ang-2, in concert with VEGF) or may act on local stromal, immune or tumor cells to influence angiogenic processes indirectly (as is the case for MMP-9, TNF-α, CXCL-12, NGAL, and IGF-1) [1,6,7,9,11,14,15,24,25,26,27,28]. For example, angiogenesis induced by TNF-α can be modulated by VEGF and FGF-2 secreted through a paracrine mechanism [9,14]. Moreover, certain factors (such as NGAL and MMP-9) can exhibit either pro- or anti-angiogenic effects, depending on the type of cancer [17]; thus, NGAL reduced angiogenesis by blocking VEGF production in a model of advanced pancreatic cancer [29,30] while it increased VEGF levels and promoted angiogenesis in breast cancer [31,32]. MMP-9 enhances EC growth in vitro [26] but can also inhibit EC migration, proliferation, and tube formation through its ability to convert plasminogen into the anti-angiogenic factor angiostatin [33,34]. In solid tumors, MMP-9 can release VEGF and FGF-2 sequestered in the extracellular matrix, which in turn activate tumor-associated ECs [35,36].

Chronic lymphocytic leukemia (CLL) is the most frequent leukemia in the Western world, where it accounts for ~30% of cases [37]. The disease is caused by the accumulation of CD5^+^CD19^+^ B lymphocytes in the peripheral blood, bone marrow (BM), and lymphatic tissues [38]. CLL develops slowly, and only a small subpopulation of malignant cells proliferate in the BM, lymph nodes (LNs), and spleen [38]. CLL is clinically heterogenous [38]: some patients have an indolent course and are not treated, while others have a more aggressive disease that requires early treatment and have shortened survival [38]. Cytogenetic abnormalities and molecular defects underpin CLL cell survival, proliferation and migration through the vascular endothelium and into the tissue/extravascular compartments [38,39]. At present, CLL is treated with a fludarabine-cyclophosphamide-rituximab (FCR) combination and signaling inhibitors that target B-cell receptor (BCR)-associated kinases (i.e., Bruton tyrosine kinase (BTK) inhibitors such as ibrutinib and acalabrutinib) or the antagonist of the B-cell lymphoma-2 (Bcl-2) anti-apoptotic protein (venetoclax) [40]. The combination of conventional cancer drugs with CD20 monoclonal antibodies (mAbs) (i.e., rituximab and obituzumab) is also proposed [40]. These therapies are often accompanied by non-genomic resistance or favored mutations associated with drug resistance [5,41,42,43,44,45]. Thus, novel therapeutic strategies are needed, and the identification of new drug targets in CLL is of great interest. Angiogenesis participates to the disease mechanism in CLL and can provide a rationale for novel therapeutic approaches in this context [2,46,47,48]. VEGF, Ang-2 and MMP-9 are involved in CLL angiogenesis. These factors do not act solely through angiogenic pathways by contributing to the progression of CLL because they also influence the survival, proliferation and migration of CLL cells themselves [2,48,49,50,51]. Here, we review (i) the expression profiles of VEGF, Ang-2, MMP-9, and other factors with potential pro-angiogenic activity in CLL (e.g., FGF-2, TNF-α, CXCL-12, CXCL-2, NGAL, IGF-1, progranulin, and angiogenin), (ii) recent advances in understanding these factors’ respective roles in CLL and their relationship with CLL angiogenesis, and (iii) current strategies for treating CLL, with a focus on the ongoing (pre)clinical development of effective, selective agents intended to target these molecules and block CLL progression.

## 2. Angiogenesis in CLL: Involvement of VEGF, Ang-2, and MMP-9

Under physiological conditions, the vasculature in the BM orchestrates hematopoiesis and angiogenesis [52,53]. Similarly, LNs promote angiogenesis and increased vascularization [54]. Abnormally prominent angiogenesis has been documented in biopsies of BM and LNs from patients with CLL [2,46,47,55,56,57,58,59,60,61,62,63,64]. The microvessel density (MVD) was assessed as a marker of angiogenesis. Several studies showed that relative to healthy individuals, the MVD is significantly higher in the BM of patients, with CLL indicating the development of new blood vessels [47,55,56,57,58,59,60,61]. Enhanced angiogenesis in the BM appears to be related to the disease stage and is predictive of a poor clinical outcome in CLL [49,56,57,60,64,65,66,67]. Moreover, vascularization of the BM is accompanied by the adhesion of CLL cells to the stroma (including ECs, fibroblasts, and macrophages) [68,69,70,71]: both activated stromal cells and activated CLL cells are able to produce VEGF, Ang-2, and MMP-9 [56,57,70,72,73], which in turn can modulate neovascularization in different ways [3,69,70,74,75,76] (Table 1). For instance, the interaction between CLL cells and BM fibroblasts induces VEGF upregulation and TSP-1 downregulation in CLL cells [68,69]; both CLL-derived VEGF and Ang-2 increase EC tube formation in vitro [74]; and MMP-9 produced by CLL cells and stromal macrophages increases VEGF production by CLL cells, which enhances EC proliferation [70]. Furthermore, advanced CLL is associated with elevated plasma/serum concentrations of VEGF, Ang-2, and MMP-9 (expressed by circulating blood CLL cells) [57,64,65,77,78,79,80,81] (Table 1), and so these factors can be used as markers of MVD in the BM [57,74,77,79,82,83,84,85,86]. In contrast, circulating levels of FGF-2 and angiogenin do not appear to reflect the MVD in the BM [57,87]. Increased vascular density is also observed in the LNs of CLL patients [2,62,63] and is associated with high levels of VEGF and interleukin (IL)-6 [63].

CLL cells and tumor-associated stromal cells also produce and secrete FGF-2, TNF-α, CXCL-12, CXCL-2, NGAL, IGF-1, progranulin, and angiogenin [88,89,90,91,92,93,94,95] (Table 1). These molecules are prognostic markers of the progression of CLL and are known (apart from angiogenin) to be involved in the functional deregulation of CLL cells (i.e., with regard to survival, proliferation, and migration) [2,51,92,93,94,96,97] (Table 1). Outside the field of CLL, these factors have been validated as pro-angiogenic factors in various inflammatory and neoplastic diseases [3,75,76,98]. Nonetheless, it is still not clear whether FGF-2, TNF-α, CXCL-12, CXCL-2, NGAL, IGF-1, progranulin, and angiogenin can contribute (directly or indirectly) to angiogenesis in CLL.

Additionally, CLL cells produce anti-angiogenic factors such as endostatin [99], adiponectin [100,101,102,103] and TSP-1 [68,90,104,105]. The level of endostatin is lower in the serum of CLL patients in advanced stages or with progressive disease while the level of FGF-2 is significantly higher in these groups of patients [99]. Adiponectin gene expression was invariably low and inversely correlated to percentages of ZAP-70^+^ and CD38^+^ cells [101,102,103], suggesting a limited (if any) role of leukemic cells in the production of circulating adiponectin levels. TSP-1 levels (mRNA, protein) were higher in low-risk CLL patients than in high-risk patients [90]. Co-culture of BM stroma with CLL cells results in an increased release of VEGF and a decreased release of TSP-1 [69]. Clinically, VEGF/TSP-1 ratio might be a predictor for the response to chemo-immunotherapy in CLL patients [106]. These examples show that the production of pro- and anti-angiogenic molecules by CLL cells is an active process in CLL, with a clear pro-angiogenic switch which, in turn, could promote BM neovascularization, CLL cell survival, and disease progression. These anti-angiogenic molecules in CLL have not been studied in this review.

**Table 1:** Soluble CLL factors: expression profiles and involvement in CLL cell functions and angiogenesis.

CLL Factor	Location	Prognostic Significance	CLL Cell Receptor	CLL Cell Processes and Angiogenesis
VEGF	- Plasma/serum [2,55,57,62,64,65,74,77,80,84,107,108,109,110] - CLL cells (mRNA, protein) [62,80,90,111,112] - BM stroma (protein) [57,68,113,114,115] - BM MSCs (protein) [68,71,114] - LN stroma (protein) [63]	Associated with a poor prognosis [57,77]	VEGF-R2 [2,64,113,116]	- EC tube formation [74] - Survival (STAT3/Mcl-1) [80,117,118,119,120,121] - Inhibition of MMP-9-mediated migration (STAT1) [122] - Survival upon association with VLA-4 (FAK/AKT) [123]
Ang-2	- Plasma/serum [57,65,74,84] - CLL cells (mRNA, protein) [83,90,124,125] - BM stroma (protein) [57,124]	Associated with a poor prognosis [57,74,83,84,85]	Tie-2 [126]	- EC tube formation [74] - Survival [74,126]
MMP-9	- Plasma/serum [78,81,127] - CLL cells (mRNA, protein) [79,86,92,127,128] - BM macrophages (protein) [70]	Higher levels in stage C disease [79,86]	CD44/VLA-4 [50]	- EC proliferation [70] - Survival (Lyn/STAT3/Mcl-1) [50] - Migration inhibition [129,130]
FGF-2	- Plasma/serum [2,55,57,65,77,85,88,89,99,109] - CLL cells (mRNA, protein) [88,89,90] - BM stroma (protein) [68]	Associated with a poor prognosis [2,55,57,65,77,80,85,88,89,96,99,102,109,131]	FGF-R3 [132]	- Survival (ERK1/2, STAT3, c-Src, Mcl-1) (Bcl-2) [2,68,96]
TNF-α	- Plasma, serum [91,97,133,134,135,136] - CLL cells (mRNA, protein) [91,133,137,138] - BM stroma (mRNA, protein) [133,137,138]	Associated with a poor prognosis [91,97,133,134,135,136]	TNF-R1 [97]	- Survival [137,138,139,140], (NF-κB) [97], (Bcl-2, Mcl-1) [97,141,142]
CXCL-12	- Serum [143] - BM stroma (mRNA, protein) [71,144]	Advanced Rai stage [143]	CXCR4 [145,146,147]	- Survival (ERK1/2, STAT3) [148,149], (SYK) [150], (ZAP70/MEK/ERK1/2) [151] - Migration (Ca2+ flux) [145], (PI3K) [152,153], (SYK) [150], (ERK1/2) [148,149], (ZAP70/MEK/ERK1/2) [151] - Survival [154], and migration (ZAP70) [155] upon binding to CD38 - Adhesion and migration dependent on VLA-4 and VEGF [156] - Migration upon VLA-4 activation (JAK2/BTK/RhoA) [157] - Secretion of CLL cell MMP-9 upon adhesion (ERK1/2/c-Fos) [128]
CXCL-2	- Plasma [73,158] - CLL cells (protein) [73,158]		CXCR2 [159]	- Survival [73,158]
NGAL	- Serum [92] - CLL cells (mRNA, protein) [79,92]	Associated with a poor prognosis [92]	SLC22A17/ NGAL-R [92]	- Survival (Src/Stat3/Mcl-1) [92]
IGF-1	- Serum/plasma [93,160,161] - CLL cells (protein) [93]		IGF1-R [93,162]	- Survival (Bcl-2) [93], (PI3K/AKT, MAPK) [162]
Progranulin	- Serum [94,148,163,164,165] - CLL cells (mRNA, protein) [84,165] - BM MSCs & LN macrophages (mRNA, protein) [94,165]	Associated with a poor prognosis [84,94,165]	TLR-9 [94]	- Survival [94]
Angiogenin	- Plasma, serum [87,95] - CLL cells (mRNA, protein) [95,103]	A prognostic marker [87]	Unknown	- Unknown

Ang-2, angiopoietin-2; BM, bone marrow; BTK, Bruton tyrosine kinase; CLL, chronic lymphocytic leukemia; CXCL, CXC chemokine ligand; CXCR4, CXC chemokine receptor-4 specific for CXCL-12; EC, endothelial cell; FAK, focal adhesion kinase; IGF-1, insulin-like growth factor-1; LN, lymph node; MMP-9, matrix metalloproteinase-9; MSC, mesenchymal stem/stromal cell; NGAL-R, neutrophil gelatinase-associated lipocalin receptor; PI3K, phosphatidyl inositol-3 kinase; TLR-9, Toll-like receptor-9; TNF, tumor necrosis factor; VEGF-R2, vascular endothelial growth factor receptor-2; VLA-4, very late antigen-4. Concentrations of soluble factors in plasma/serum were determined using various commercial ELISA kits according to the manufacturer’s instructions.

## 3. Expression Profiles and Functions of VEGF, Ang-2, MMP-9, FGF-2, TNF-α, CXCL-12, CXCL-2, NGAL, IGF-1, Progranulin, and Angiogenin in CLL

The interactions between CLL cells and the surrounding stromal cells in the BM and LN microenvironments favor CLL cell survival, proliferation, and migration via the production of endocrine or paracrine factors [63,69,70,71,72,76]. In this section, we review current knowledge about the expression profiles and roles of VEGF, Ang-2, MMP-9, FGF-2, TNF-α, CXCL-12, CXCL-2, NGAL, IGF-1, progranulin, and angiogenin in CLL (Table 1).

### 3.1. VEGF

High serum or plasma concentrations of VEGF were reportedly higher in CLL patients than in healthy controls and defined a subset of CLL patients with a poor clinical outcome [64,65,74,77,80,84,109] (Table 1). The plasma VEGF concentration decreased after fludarabine-based treatment [110]. In CLL, VEGF is expressed by primary CLL tumor cells and BM MSCs [57,64,68,112,114] (Table 1). Hypoxia is a crucial parameter in angiogenesis and tumor development [166]. Under hypoxic conditions, mRNA and protein levels of VEGF are elevated in CLL cells [62]. In particular, CLL cells synthesize and release VEGF_165_ [80,111]. The main receptor for VEGF_165_ is VEGF-R2, which is usually overexpressed in CLL cells [116]. CLL patients with high VEGF-R2 levels have marked lymphocytosis, severe anemia, and a shorter survival time [116]. Accordingly, autocrine VEGF drives CLL cell survival and prevents drug-induced apoptosis [117,118] by interacting with VEGF-R2 and by upregulating STAT3 and the pro-survival protein Mcl-1 (a member of the BCL-2 family) [117,119,120,121] (Table 1). Relative to CD38^-^ CLL cells, CD38^+^ CLL cells from patients with a poor prognosis overexpress VEGF, VEGF-R2 and Mcl-1, and this overexpression is associated with apoptosis resistance [80,120] (Table 1). Gehrke et al. reported that BM stromal VEGF (rather than CLL cell VEGF) is involved in the survival of CLL cells [114]. VEGF/VEGF-R2 interaction downregulates MMP-9 expression (via STAT1 activation) and consequently inhibits CLL cell migration [122] (Table 1). Surface VEGF-R2 physically associates with the integrin very late antigen-4 (VLA-4/α4β1) [51], and engagement of the VEGF-R2/VLA-4 complex by VEGF activates a survival pathway that involves the phosphorylation and activation of focal adhesion kinase (FAK) and AKT [123] (Table 1).

### 3.2. Ang-2

CLL patients with a poor clinical outcome have elevated levels of serum/plasma Ang-2 [57,65,74,84] (Table 1). Binet stage B and C cases have higher plasma Ang-2 concentrations than Binet stage A cases, which suggests a link between Ang-2 and CLL progression [84]. In particular, an elevated plasma Ang-2 concentration was reported in patients with an unmutated sequence for the variable region of the immunoglobulin heavy chain (IGVH) and high expression of ZAP-70 and CD38 and in patients with an intermediate or high cytogenetic risk [57,65,74,84]. Accordingly, elevated mRNA expression of Ang-2 in blood CLL cells is associated with unmutated IGVH genes and shorter progression-free survival [83,90,124,125]. BM stromal cells are also able to produce Ang-2 [57] (Table 1). By binding to its receptor Tie-2 [126], Ang-2 favors CLL cell survival [74,126] (Table 1). However, the signaling transduction pathways have not been characterized.

### 3.3. MMP-9

Plasma/serum MMP-9 concentrations are significantly higher in untreated early-CLL patients (stage A) than in healthy controls [78,81,127] but fall to near-control levels in patients in remission [81,92] (Table 1). In contrast to resting B lymphocytes, CLL cells (stage A, according to the Binet classification) synthesize and secrete the inactive zymogen form of MMP-9 (proMMP-9) [79,127,128] (Table 1). Moreover, the highest levels of intracellular MMP-9 are associated with advanced (Binet stage C) disease and with poor overall survival [79]. (Pro)MMP-9 binds to its docking receptors VLA-4 and CD44, which are overexpressed on CLL cells [50] (Table 1). By binding to VLA-4 in concert with CD44, MMP-9 induces an intracellular signaling pathway that favors the survival of CLL blood cells [50] (Table 1). This pathway consists of LYN kinase activation, STAT3 phosphorylation, and Mcl-1 activation [50] (Table 1). As a consequence, MMP-9 bound to both VLA-4 and CD44 impairs CLL cell migration [129,130] (Table 1). As mentioned in Section 2, MMP-9’s interaction with CLL cells increased the expression and secretion of VEGF and decreased TSP-1 expression [70]; thus, MMP-9-primed CLL cells significantly enhanced VEGF-mediated EC proliferation [70] (Table 1).

### 3.4. FGF-2

Circulating (plasma/serum) levels of FGF-2 are higher in CLL patients than in healthy controls [2,55,57,65,77,80,85,88,89,96,99,102,109] (Table 1). Elevated plasma levels of FGF-2 in CLL patients decrease after fludarabine-based treatment **[110]**. CLL blood cells and BM stromal cells synthesize and release FGF-2 [68,88,89,90] (Table 1). Protein and mRNA levels of FGF-2 inside CLL cells are correlated with the clinical CLL stage [57,88,131]. An in vitro interaction between CLL cells and BM stroma markedly increased FGF-2 secretion and decreased TSP-1 secretion, leading to CLL cell escape from spontaneous and drug-induced cell death [68]. By binding to its receptor FGF-R3 [132], FGF-2 increases CLL cell survival through the activation of ERK1/2 and c-Src kinases, STAT3 phosphorylation, and activation of Mcl-1 and Bcl-2 (another major member of the pro-survival BCL-2 family) [2,68,96] (Table 1).

### 3.5. TNF-α

Elevated levels of soluble TNF-α and its receptor (TNF-R1) are detected in the sera of patients with CLL and are associated with a poor clinical outcome [91,97,133,134,135,136] (Table 1). In particular, high serum concentrations of TNF-α are more likely to harbor high-risk chromosome abnormalities and advanced disease [134]. Accordingly, a simultaneous increase in serum TNF-α and IL-10 levels was observed in a high-risk CLL subgroup with a shorter 3-year treatment-free survival time and a higher leukocyte count [167]. TNF-α is expressed constitutively by CLL cells and BM stromal cells [91,133,137,138] (Table 1). Aberrant high expression of TNF-R1 has been observed in the LNs of CLL patients [97].

TNF-α activates the transcription factor NF-κB, which in turn regulates TNF-α production [10]. In CLL, NF-κB is activated to a variable degree, regardless of the disease stage or treatment status [97]. For instance, NF-κB is activated in CLL cells with unmutated IGHV genes upon exposure to TNF-α [168]. TNF-α has been shown to act as an autocrine and paracrine growth factor that induces CLL cell proliferation in vitro [137,138,139] (Table 1). TNF-α favors cell survival and proliferation of CLL cells by upregulating Bcl-2 and Mcl-1 [97,141,142]. Accordingly, stimulation of TNF-R1 with TNF-α enhanced NF-κB activity and CLL cell survival [97] (Table 1). More recently, NF-κB signaling has been characterized as comprising two independent but interlinked signaling pathways [10]: the canonical or classical pathway mediated by the action of the RelA/p50 subunits, and the non-canonical or alternative pathway that is dependent on activation of the RelB subunit associated with p50 or p52 [10]. RelA binding complexes are constitutively active in blood CLL cells, and their activation is STAT3-dependent [169]. Activities of both RelA and RelB were detected in CLL cells isolated from BM aspirates and were shown to confer survival advantages on CLL BM cells [140] (Table 1). RelB activity enhances cell sensitivity to proteasome inhibitors but not to fludarabine [140].

### 3.6. CXCL-12 and CXCL-2

CXCL-12 (also known as stromal-derived factor-1) is expressed by BM stromal cells in CLL patients [71,144] (Table 1). A significant correlation was observed between the serum CXCL-12 level and an advanced Rai stage [143]. CXCL-12’s receptor (CXCR4) is expressed on many cell types [170] and is a key chemokine receptor on CLL cells [146,147]. Relative to normal B cells, CLL cells display higher levels of total and surface CXCR4 [145]. Elevated CXCR4 expression is associated with a poor prognosis, resistance to FCR therapy [171], and a greater risk of lymphoid organ infiltration [172]. Overexpression of CXCR4 on CLL cells is associated with greater functional responses to CXCL-12 [39,147]. The CXCL-12/CXCR4 axis exerts at least two major effects on CLL cells: the induction of survival signals and cell migration toward the stroma (Table 1). CXCL-12/CXCR4-mediated signaling involves Ca^2+^ flux [145,151] and the activation of PI3K [152,153], SYK [150], ERK1/2 [148,149], ZAP70, MEK, ERK1/2 [151], and STAT3 [149] (Table 1). The surface expression of CXCR4 is strongly associated with that of CD38, VLA4, MMP-9, and BCR [51]. Accordingly, CXCL-12/CXCR4 signaling in CLL cells can be modulated by the BCR, CD38, VLA-4, and VEGF [51,154,155,156]. BCR activation upregulates the expression of CXCR4 in CLL cells [173], and treatment of CLL cells with ibrutinib (a BTK inhibitor) is followed by downregulation of surface CXCR4 expression and inhibition of CXCL-12/CXCR4 downstream signaling [174]. The physical interaction between CXCR4 and CD38 increases the intensity of the CXCL-12-mediated signals involved in the survival [154] and migration of ZAP70^high^ CLL cells [155]. Both VEGF (through binding to VEGF-R) and VLA-4 are involved in the CXCL-12-dependent motility of CLL cells towards and through the endothelium [156] (Table 1). CXCL-12 induces an active VLA-4 conformation on CLL cells; this results in the involvement of VLA-4 in CXCR4-dependent CLL cell migration and adhesion to the stroma via the JAK2/BTK/RhoA signaling cascade [157] (Table 1). Consequently, MMP-9 expression and release are upregulated via an ERK1/2/c-Fos signaling pathway and are involved in CLL cell migration [128] (Table 1).

A significant elevation in the plasma CXCL-2 (also known as monocyte inhibitory protein-2α) concentration is observed in patients with CLL versus healthy controls [73,158] (Table 1). CXCL-2 is produced by various cell types, including stromal cells, ECs, and tumor cells [73]. CXCL-2 is strongly expressed by CLL cells when co-cultured with BM stromal cells [73,158] (Table 1), and its expression appears to be correlated with sustained CLL cell survival in vitro [73,158] (Table 1).

### 3.7. NGAL

NGAL is a glycosylated protein from the lipocalin family. It exists as a monomer, a homodimer, and a disulfide-linked heterodimer bound to proMMP-9 [17,175]. NGAL concentrations (whether free or complexed to MMP-9) are elevated in the serum of CLL patients at diagnosis [92] (Table 1). After treatment (and regardless of the therapeutic regimen), serum NGAL levels normalized in CLL patients in remission but not in relapsed patients [92]. Cultured CLL cells express and release NGAL and the NGAL/MMP-9 dimer [79,92] (Table 1). The NGAL receptor (NGAL-R) belongs to the SLC22 family of organic ion transporters [51,176]. The NGAL-R is absent or weakly expressed in normal peripheral blood cells but is strongly expressed by CLL cells from treatment-naive patients, and its expression is associated with the clinical prognosis [92] (Table 1). Surface NGAL-R physically associates with CD38 [177], and patients with progressing CLL showed a time-dependent increase in NGAL-R/CD38 levels [177]. In treated CLL patients who achieved clinical remission, NGAL-R/CD38 levels were decreased and fell to baseline levels [92,177]. Upon binding to NGAL, NGAL-R provides CLL cells with an SRC/STAT3/Mcl-1-dependent survival signal [92] (Table 1).

### 3.8. IGF-1

There are conflicting reports on circulating IGF-1 levels in CLL. Two studies have found that plasma and serum levels of IGF-1 are higher in CLL patients than in age-matched, healthy controls [93,160] (Table 1). However, another study found the opposite: lower serum levels of IGF-1 in patients with CLL (Binet stage A) than in a control group, and no significant correlation between serum IGF-1 levels and clinical and hematological variables (including the Rai stage) [161] (Table 1). In general, the relationship between circulating IGF-1 concentrations and various factors (including genetic factors and age) is multifaceted and may influence the interpretations of research results [178]; this might explain the contradictory findings on circulating IGF-1 levels in CLL. The IGF-1 receptor (IGF-1-R, also known as CD221) is a receptor tyrosine kinase primarily activated by IGF-1/-2 [179]. Most CLL cells secrete IGF-1 and express IGF-1-R [93,162] (Table 1). IGF-1 expression is lower in CLL cells from patients with del 13q than in cells of patients with high-risk genetic features [162]. IGF-1-R overexpression was found in all CLL subsets (13q, Tri12, 11q, 17p) [162]. Upon IGF-1 stimulation, CLL cells activate the PI3/AKT, MAPK, and Bcl-2 pathways [93,162] (Table 1).

### 3.9. Progranulin

The serum progranulin concentration is elevated in CLL patients with an advanced stage of disease [94,148,163,164,165] and is an independent predictor of disease progression and overall survival in CLL [84,94,165] (Table 1). Progranulin is expressed by CLL cells and found upregulated in ZAP70^+^CD38^+^ CLL cells (associated with a poor prognosis), relative to ZAP70^-^ CD38^-^ CLL cells [84,165]. This factor is also expressed by BM MSCs and LN macrophages in CLL [94,165] (Table 1). Progranulin co-activates Toll-like receptor-9 (TLR-9) which is strongly expressed by CLL cells and can convey proliferative and survival signals [94] (Table 1).

### 3.10. Angiogenin

Serum angiogenin levels in CLL patients are similar to those measured in healthy controls [87,95] (Table 1). However, prolonged progression-free survival appears correlated with a high angiogenin level (≥330 ng/mL), which might therefore be predictive of the clinical outcome in patients with early-stage CLL [87] (Table 1). In general, angiogenin is expressed by most ECs, fibroblasts, and hematopoietic cells [11,180]. Cultured blood CLL cells (stage A) express endogenous angiogenin (both mRNA and protein are detected) and release it into the circulation [95]. Accordingly, angiogenin was found to be significantly higher in CLL patients than in controls and its level increased with the clinical stage [103]. In various experimental tumor cell models, angiogenin regulates cell proliferation, migration, and adhesion by activating the SAPK/JNK, ERK1/2, and PI3K/AKT pathways in various cells and under different conditions [11,180]. The angiogenin receptor plexin-B2 [181] and angiogenin’s functions have not been identified in primary CLL cells; preclinical studies of angiogenin’s role(s) in this setting are therefore needed.

## 4. Preclinical and Clinical Trials of Drugs That Target Cell Survival Factors and Their Receptors in CLL

A number of preclinical and clinical trials in CLL have targeted VEGF/VEGF-R, MMP-9/CD44/VLA-4, CXCL-12/CXCR4, TNF-α/TNF-R, and IGF-1/IGF-1-R (Table 2). In contrast, Ang-2, FGF-2, NGAL, progranulin, and angiogenin have not been investigated as targets in the context of CLL.

### 4.1. VEGF/VEGF-R2 Inhibitors

Several agents targeting the VEGF/VEGF-R axis are currently in clinical development for the treatment of cancers. These agents include VEGF-R mAbs and inhibitors that block signaling through VEGF-R, and anti-VEGF-R2 chimeric antigen receptor (CAR)-T cell constructs [21,40,51,208,209,210]. Two human anti-VEGF Abs (bevacizumab and ramucizumab) have been approved by the US Food and Drug Administration (FDA) for the treatment (alone or combined with other drugs) of certain cancers [211,212]. In vitro, bevacizumab exhibits pro-apoptotic effects on CLL cells [182]: it triggers leukemia cell death, with activation of caspases 3/9, overexpression of the proapoptotic factors Bak and Bad, and downregulation of Mcl-2 and AKT [182] (Table 2). The combination of bevacizumab with rituximab, alemtuzumab, or rapamycin significantly increased in vitro CLL cell death, relative to each drug alone [90,182] (Table 2). Bevacizumab monotherapy did not have significant clinical efficacy in patients with refractory/relapsed (R/R) CLL [183] (Table 2). Moreover, a combination of bevacizumab with FCR did not improve outcomes in patients with relapsed CLL, when compared with patients treated with FCR [184] (Table 2). Moreover, the response to bevacizumab appeared to be weak, and resistance soon appeared [212,213]. However, the results of a recent Phase II randomized trial showed that the addition of bevacizumab to chemoimmunotherapy (pentostatin, cyclophosphamide, and rituximab) in CLL was well tolerated and appeared to prolong progression-free and treatment-free survival of patients with progressive but previously untreated CLL [185] (Table 2).

### 4.2. MMP-9/CD44/VLA4 Inhibitors

The initially developed anti-enzyme therapies targeted MMP-9s’ catalytic activity and thus sought to inhibit tumor progression **[214,215,216]**. The treatment failures observed with MMP-9 inhibitors in Phase III clinical trials in patients with solid tumors might be due to their lack of selectivity and specificity for MMP-9, which leads to undesired off-target effects **[214,215,216,217]**. More recent therapeutic strategies include DNA/RNA aptamers and peptides that block MMP-9’s interactions with its cell surface receptors CD44 and VLA-4 and function-blocking mAbs that bind to CD44 and VLA-4 [189,216,218,219,220,221]. The peptide A6’s binding to CD44 results in the inhibition of migration and metastasis of solid tumor cells and the modulation of CD44-mediated cell signaling [186]. A6 has shown efficacy and an excellent safety profile in Phase Ia, Ib, and II clinical trials in patients with solid tumors [186]. In vitro, A6 induces the death of ZAP-70^+^ CLL cells [186] (Table 2). Similarly, the CD44-binding peptide P6 (which binds to the hemopexin domain of MMP-9) impairs the adhesion and migration of CLL cells [187] (Table 2). These peptides have not yet been tested in Phase I trials on patients with CLL. When considering the anti-CD44 mAbs in preclinical or clinical development as cancer therapies [189,218,219], the humanized anti-CD44v6 mAb RG7356 has been shown to induce the in vitro caspase-dependent death of ZAP-70^+^ CLL cells from patients with a poor prognosis [188] (Table 2). Administration of RG7356 to immunodeficient mice engrafted with human CLL cells resulted in complete clearance of the latter [189,190]. The FDA-approved anti-CD49d mAb natalizumab has emerged as a potential treatment for cancer [222,223,224]. In vitro, natalizumab inhibits the VLA-4-dependent migration of CLL cells [191] (Table 2). These approaches warrant further investigation as possible treatments for CLL.

### 4.3. TNF-α/TNF-R Inhibitors

Several classes of TNF-α inhibitors are available, including anti-TNF Abs, inhibitors of TNF expression (such as thalidomide and its analog lenalidomide), soluble TNF-Rs (such as etanercept), and inhibitors of TNF-α-induced signaling pathways (such as NF-κB blockers) [8,10,12]. To date, attempts to treat hematologic malignancies (including CLL) with anti-TNF-α Abs have not produced objective therapeutic anti-cancer responses. In fact, TNF-α Abs bind both soluble (s) TNF-α and transmembrane TNF-α, and so the targeted binding of Abs to leukemia cells is largely neutralized by sTNF-α [15,225]. Thalidomide and lenalidomide combine immunomodulatory and anti-angiogenic effects by inhibiting NF-κB activity, TNF-α expression, and (to a lesser extent) FGF-2 and VEGF expression in various tumor cell types [64,226]. A large number of clinical trials have studied the use of thalidomide or lenalidomide in treatment-naïve patients and patients with R/R CLL, either as single agents or in combination with chemotherapy (rituximab, ibrutinib, obinutuzumab, etc.) [194,195,227,228,229,230,231,232,233]. The onset of efficacy was slow, and toxicity limited the use of thalidomide or lenalidomide as a single agent or combined with chemotherapy [195,234,235,236]. A lenalidomide rituximab combination is currently being evaluated in a Phase I trial for the treatment of CLL [194,195] (Table 2). Etanercept inhibits the biological activity of soluble TNF-α [138,237]. In patients with refractory hematological diseases (including CLL), etanercept was found to be well tolerated and not associated with an overt increase in infectious episodes [225,238]. While rituximab is ineffective in relapsed CLL with del 17p [239], the combination of rituximab and etanercept was well tolerated and demonstrated clinical activity in relapsed CLL patients without del 17p [197,198] (Table 2). However, the addition of etanercept did not improve the clinical response rate beyond that expected with thrice weekly single-agent rituximab [197,198,232] (Table 2).

### 4.4. CXCL-12/CXCR4 Inhibitors

With a view to blocking the CXCL-12/CXCR4 axis, small chemical inhibitors (such as plerixafor and BKT140), RNA oligonucleotides, and blocking mAbs have been investigated in various cancer settings [149,240,241]. In CLL, several clinical trials combined a CXCR4 antagonist with conventional cytotoxic agents (i.e., bendamustine, fludarabine, cyclophosphamide, and lenalidomide) or mAbs (i.e., rituximab and alemtuzumab) [233,240,241,242]. CXCL-12 targeting was achieved through the use of RNA oligonucleotides; for example, NOX-A12 inhibits CLL-cell migration in vitro and sensitizes CLL cells to cytotoxic agents [199] (Table 2). A combination of bendamustine, rituximab, and NOX-A12 has been tested in a Phase II trial in relapsed CLL patients [200]; the treatment was well tolerated and did not result in more toxicity than the two-drug bendamustine–rituximab combination [201] (Table 2). In a Phase I trial of plerixafor plus lenalidomide in previously treated CLL patients, the most common grade 3/4 toxicities were anemia, neutropenia, and thrombocytopenia [204] (Table 2). Another multicenter Phase I study of plerixafor and rituximab in patients with R/R CLL showed that the combination was well tolerated, with CLL cell mobilization in the blood: maximum responses could be still detected several months after completion of the course of treatment [205,206] (Table 2). Thus, the combination of a CXCR4 antagonist with conventional agents might help to mobilize and eliminate residual CLL cells. Although the action of therapeutic mAbs against leukocyte CXCR4 is complicated by the protein’s conformational heterogeneity, Abs that inhibit the CXCL-12/CXCR4 axis are advancing well through the clinical development process [243]. For example, the fully human IgG4 anti-CXCR4 mAb ulocuplumab induces the death of primary CLL cells in vitro through a reactive oxygen-species-dependent pathway [207] (Table 2). Ulocuplumab’s safety and tolerability in patients with CLL have been assessed in a Phase I trial (Table 2), the results of which have not yet been published.

### 4.5. IGF-1/IGF-1-R Inhibitors

A variety of IGF-1/IGF-1R inhibitors have entered clinical development in the cancer field [244,245,246,247,248], including IGF-1-R tyrosine kinase inhibitors (including sorafenib) and mAbs against IGF-1-R and IGF-1. Most of these clinical trials failed to evidence clinical benefits in the trial population as a whole [248]. Sorafenib is a broad-spectrum kinase inhibitor that targets the RAF/MEK/ERK pathway and receptor tyrosine kinases (RTKs) such as IGF-1-R and VEGF-R [249,250]. It is an effective first-line therapy in advanced hepatocellular carcinoma [250]. In CLL, sorafenib inhibits in vitro leukemic cell survival by downregulating IGF-1-R expression and phosphorylation and thus counteracting IGF-1’s binding to its receptor [162] (Table 1).

### 4.6. Inhibitors Targeting Ang-2, FGF-2, CXCL-2, Angiogenin, and NGAL in Other Tumors

A number of drugs targeting Ang-2/Tie-2 are in various stages of (pre)clinical development or are currently being used to treat cancer [180,251,252,253]. These drugs include CVX060 (two peptides that bind Ang-2 with high affinity and specificity, covalently fused to a scaffold antibody) and the anti-Tie-2 nesvacumab (REGN910) alone or in combination with VEGF inhibitors, and dual inhibitors of Ang-2 and VEGF (namely the mAbs vanucizumab and RG7716/faricimab) [180,251,252,253]. Tie-2-blocking Abs are currently being trialed on patients with acute myeloid leukemia [253,254]. Many clinical trials in an indication of solid tumors are underway for FGF ligand traps, FGF-R kinase domain inhibitors, and mAbs against FGF-R [21,255,256,257]. The recent RADICAL Phase IIa trial (NCT01791985) of AZD4547 (a potent, selective chemical inhibitor of FGF-R1/-2/-3) in endocrine-resistant breast cancer gave encouraging results [258]. Preclinical studies have identified inhibition of CXCL-2/CXCR2 as promising therapeutic strategy for inhibiting tumor progression and metastasis: developed drugs include small inhibitors targeting CXCR2 (such as AZD5069 and reparixin), CXCL-2 expression (such as miRNA MIR-532-5p) and function (such as inhibitors of signaling pathway), and anti-CXCL-2 mAbs [259]. Angiogenin exhibits ribonucleolytic activity [260]. Various angiogenin inhibitors (including enzyme inhibitors, mAbs, siRNAs, and soluble binding proteins) inhibit tumor growth in various animal models [11,180,261]; further clinical trials of angiogenin are needed. In preclinical studies, NGAL inhibitors (which interfere with NGAL activity in neoplastic and/or tumor stromal cells) include mAbs against NGAL/NGAL-R and small, selective siderophore inhibitors [262]. All these drugs might be effective in the treatment of CLL and thus warrant investigation. There are currently no (pre)clinical trials targeting progranulin in the field of cancer.

## 5. Conclusions and Perspectives

**Figure 1:**
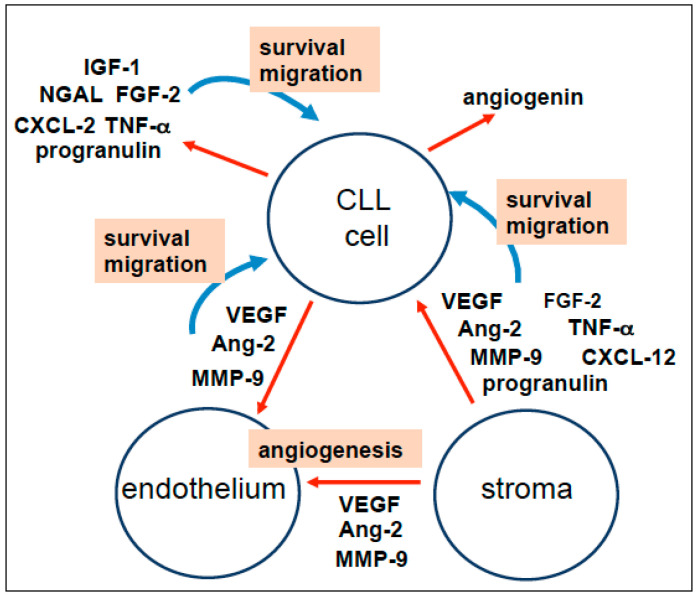
A schematic view of autocrine and paracrine loops between CLL cells, the stroma (fibroblasts, macrophages, and other MSCs), and the endothelium. Activated leukemia cells and stromal cells produce VEGF, Ang-2, and MMP-9, which induce the angiogenic process (i.e., EC migration and proliferation). Furthermore, VEGF, Ang-2, MMP-9, FGF-2, TNF-α, CXCL-2, CXCL-12, NGAL, and progranulin act as autocrine and/or paracrine inducers of CLL cell survival and migration. The functions of the angiogenin produced by CLL cells have not been characterized. In turn, activated endothelium may produce growth factors or chemokines (such as Ang-2, progranulin, angiogenin, IGF-1, MMP-9, VEGF, TNF-α, and CXCL-12) [28,73,216,263,264,265,266,267,268] that can affect leukemic and stromal cells.

Here, we reviewed published data on how interactions with the tumor microenvironment influence the angiogenic process and the survival and growth of CLL cells (Figure 1). In particular, knowledge of the functional significance of circulating factors in the relationship between angiogenesis and CLL cells might drive the development of novel therapeutics in this field.

As shown in Figure 1, the dynamic crosstalk between leukemic cells, ECs, and stromal cells (including fibroblasts, macrophages, and other MSCs) in the BM and LNs of CLL patients stimulates angiogenesis and thus promotes the survival, proliferation, and migration of leukemic cells. It remains to be seen whether this is also true for secondary lymphoid tissues (such as the spleen and other extranodal sites) in CLL. Moreover, additional research is needed to determine whether BM MSCs (such as dendritic cells, adipocytes, osteoclasts, and osteoblasts) can provide signals and thus contribute to the angiogenic response and the functional deregulation of CLL cells [4,269]. For instance, BM adipocytes release TNF-α, which contributes to the growth and migration of multiple myeloma cells [270], modifies the pharmacokinetics of chemotherapy, and drives the proliferation of acute lymphocytic leukemia T cells [271].

In the CLL BM, interactions between all cell types result in the secretion of (and responses to) soluble factors [4,5,52,272,273]. VEGF, Ang-2, and MMP-9 released by leukemic cells and stromal cells induce the angiogenic process by stimulating the migration and proliferation of ECs (Figure 1). In turn, new vessels are likely to contribute to the initiation and maintenance of a favorable microenvironment for leukemic and stromal cells by providing nutrients and oxygen. Moreover, VEGF, Ang-2, and MMP-9 can elicit survival, adhesive, and/or migratory states in CLL cells (Figure 1), as do other circulating factors discussed in this review (FGF-2, TNF-α, CXCL-12, CXCL-2, NGAL, IGF-1, and progranulin; Figure 1). It remains to be determined whether FGF-2, TNF-α, CXCL-12, CXCL-2, NGAL, IGF-1, and progranulin are, directly and/or indirectly, involved in the induction of angiogenesis and should therefore be considered as pro-angiogenic factors. Under physiological conditions, ECs produce various growth factors and chemokines, including Ang-2, progranulin, angiogenin, IGF-1, MMP-9, VEGF, TNF-α, CXCL-12, and CXCL-2 [28,73,216,263,264,265,266,267,268]; it is likely that activated CLL ECs produce these molecules, which could in turn affect leukemic and stromal cells. In summary, the ability of these factors to stimulate CLL angiogenesis and/or favor CLL cell motility and survival suggests that these proteins might be excellent therapeutic targets in CLL.

In addition to the circulating factors reviewed here, other components in the CLL microenvironment might have a crucial role in CLL pathogenesis by interfering with angiogenesis and CLL cell functions. The growing list of pro-angiogenic/survival factors includes IL-6, IL-8, leptin, placental growth factor, TGF-β, platelet-derived growth factor B, and other chemokines (such as CXCL-13, CXCL-9/-10/-11, CCL-19, CCL-21) [21,63,71,73,76,147,274,275]. The elevated expression of these factors in blood, BM, and other lymphoid tissues adds additional complexity to CLL disease.

Patients with high-risk CLL disease (~25% of the total) are either refractory to today’s front-line therapies or relapse after treatment and become chemoresistant [38]. To improve clinical outcomes and immune function in CLL patients, more selective BTK and BCL-2 inhibitors are in clinical development [40,276,277]. Furthermore, a broad variety of mAbs (including mono-/bispecific Abs, CAR-T cells, and bi-CAR-T cells) constitutes an attractive therapeutic option for CLL [40,51,276,277,278,279]. In 2007, the mAb alemtuzumab (also known as Campath-1H, approved by the FDA for the treatment of CLL) was shown to interfere with the CLL angiogenic process [82,280]. Alemtuzumab targets the CLL cell surface antigen CD52 and its soluble form (sCD52) [281]. High sCD52 levels are associated with a significantly shorter time to first treatment, and the sCD52 level falls (along with decreases in LN size) following ibrutinib therapy [280]. A marked decrease in BM vascularity was observed in CLL patients who received alemtuzumab consolidation therapy after a clinical response to fludarabine induction therapy [82]. Despite alemtuzumab’s proven efficacy in the treatment of R/R CLL [282,283,284,285], the mAb led to serious infusion-related, hematologic, and infection-related adverse events and was replaced in 2020 by rituximab [236].

The soluble factors (and their receptors) discussed in this review have been validated as therapeutic targets in CLL. So far, various drugs have been developed against some of these factors (and/or their receptors) and include mAbs and small inhibitors; they have already been evaluated, alone or in combination with conventional agents (FCR, PCR, ibrutinib, bendamustine, rituximab) in clinical studies in untreated and R/R CLL: they include bevacizumab (anti-VEGF), ulocuplumab (anti-CXCR4), plerixafor (CXCR4 inhibitor), Nox-A12 (CXCL-12 inhibitor), and etanercept (soluble TNF-R) (Table 2). These phase I/II trial results are promising and should support a move into phase III. Besides its clinical efficacy, primary and acquired resistances to ibrutinib have been described in CLL [286]. Of the new selective BTK inhibitors developed in the last 10 years (including acalabrutinib, zanubrutinib, and pirtobrutinib), acalabrutinib is authorized for untreated and R/R CLL and CLL with 17p deletion [40]. In a phase III trial, zanubrutinib significantly improved response rates and delayed disease progression in patients with R/R CLL (relative to ibrutinib) and did so with less toxicity [40]. The efficacy and safety of zanubrutinib are being evaluated in treatment-naive CLL patients with and without a 17p deletion [40]. When combined with rituximab, venetoclax is now an approved standard of care for treatment- and relapsed CLL disease [40]. Three ongoing phase II trials are evaluating the venetoclax + ibrutinib combination in treatment-naive CLL patients and patients with R/R CLL, and the venetoclax + ibrutinib + obinutuzumab (anti-CD20) combination in treatment-naive CLL patients with a p53 deletion (17p-) and/or mutation (reviewed in [40]). Pirtobrutinib is undergoing clinical development as monotherapy or combination (with venetoclax and rituximab) therapy in untreated or previously treated CLL [40]. Thus, given their potential complementary activity, the combinations of angiogenic inhibitors with these new BTK inhibitors and/or venetoclax are of interest.

Angiogenesis is increased in a number of other hematological malignancies, including B cell-non-Hodgkin lymphoma (B-NHL) (including diffuse large B cell lymphoma/DLBCL, follicular lymphoma/FL, mantle cell lymphoma/MCL, marginal zone B-cell lymphoma/MZL), multiple myeloma (MM), myelodysplastic syndrome (MDS), acute myeloid leukemia (AML), acute lymphoid leukemia (ALL), and chronic myeloid leukemia (CML) [81,253,287,288,289,290,291]. Other than VEGF, Ang-2, and FGF-2, a few other molecules have so far been implicated in this process and include VEGFR-1/2, TNF-α, IL-6, IL-8, and MMP-9 [81,253,287,288,289,290,291]. Clinical studies with various anti-angiogenic agents are underway in these malignancies [21,40,288,289,291,292,293]. Several drugs targeting angiogenesis-related pathways such as VEGF mAbs (such as bevacizumab), VEGF RTK inhibitors (such as sorafenib and sunitinib), and immunomodulatory drugs (such as thalidomide and lenalidomide) have been entered in clinical trials or are already approved for the treatment of these hematological diseases [40,289,291,293,294,295,296]. For instance, phase I/II clinical trials monitoring the susceptibility of bevacizumab or sorafenib (alone or in combination with conventional chemotherapy) showed promising results in R/R AML patients, leading to current phase III trials [295,296,297]. Lenalidomide, combined with rituximab or tafasitamab (anti-CD19), is already considered an established treatment modality for patients with DLBCL, MCL, MZL, and R/R MM (for review in [40]). Likewise, the anti-angiogenic strategy, hopefully, will achieve clinical benefits for CLL patients by stopping or slowing CLL progression, counter therapeutic resistance, and thus improve clinical outcomes and quality of life for patients with CLL.

## Figures and Tables

**Table 2:** Examples of (pre)clinical studies of drugs targeting VEGF, CD44, VLA-4, TNF-α/TNF-R, CXCL-12/CXCR4, and IGF-1-R, evaluated alone or in combination as first- or second-line therapies for CLL.

Agent	Class	Preclinical Study	Clinical Study
Bevacizumab	Anti-VEGF mAb	Induces CLL cell death [90,182]	- Phase II NCT00290810, R/R CLL [183] - Phase II NCT00448019, relapsed CLL, combined with FCR [184] - Phase II NCT00816595, previously untreated CLL, combined with PCR [185]
A6 (Angstrom 6, NSC750394)	CD44 peptide inhibitor ([acetyl]-KPSSPPEE-[NH2])	Induces CLL cell death [186]	
P6	CD44 peptide inhibitor (FDAIAEIGNQLYLFKDGKYW)	Inhibits CLL cell adhesion and migration [187]	
RG7356	Anti-CD44v6 mAb	Inhibits CLL cell survival and migration [188,189,190]	
Natalizumab	Anti-CD49d mAb (VLA-4 subunit)	Inhibits CLL cell migration [191]	
Lenalidomide	Inhibits TNF-α expression	Inhibits CLL cell survival [192,193]	- Phase I NCT01446133, previously untreated CLL, combined with rituximab [194,195]
Etanercept	Soluble TNF-R neutralizes soluble TNF-α	Inhibits CLL cell proliferation [196]	- Phase II, relapsed CLL, combined with rituximab [197] - Phase I/II NCT002182, refractory CLL, combined with rituximab [198]
NOX-A12 (olaptesed pegol)	An RNA oligonucleotide that binds and neutralizes CXCL-12	Inhibits CLL cell migration [199]	- Phase II NCT01486797, refractory CLL, combined with bendamustine and rituximab [200,201]
Plerixafor (AMD3100)	CXCR4 small molecule inhibitor	Inhibits CLL cell survival and migration [149,202,203]	- Phase I NCT01373229, R/R CLL, combined with lenalidomide [204] - Phase I NCT00694590, R/R CLL, combined with rituximab [205,206]
BKT140 (BL-8040, 4F-benzoyl-TN14003, motixafortide, TF 14016)	CXCR4 peptide inhibitor	Inhibits CLL cell survival and migration [149,202]	
Ulocuplumab (BMS-936564, MDX-1338)	Anti-CXCR4 mAb	Induces CLL cell death (via ROS) [207]	- Phase I NCT01120457, previously untreated CLL, alone or combined with ibrutinib
Sorafenib (Nexavar, BAY43-9006)	Broad multikinase inhibitor, inhibits IGF-1-R expression and kinase activity	Induces CLL cell death [162]	

BKT140,N^2^-(4-fluorobenzoyl)-L-arginyl-L-arginyl-3-(2-naphthalenyl)-L-alanyl-L-cysteinyl-L-tyrosyl-N^5^-(aminocarbonyl)-L-ornithyl-L-lysyl-D-lysyl-L-prolyl-L-tyrosyl-L-arginyl-N^5^-(aminocarbonyl)-L-ornithyl-L-cysteinyl-L-argininamide, cyclic (4→13)-disulfide. FCR, fludarabine, cyclophosphamide, and rituximab; PCR, pentostatine, cyclophosphamide, and rituximab; R/R, refractory/relapsed. Plerixafor, l,1′-[1,4-phenylene bis (methylene)]-bis-1,4,8,11-tetraazacyclotetradecane; ROS, reactive oxygen species; Sorafenib, 4-[4-[[4-chloro-3-(trifluoromethyl) phenyl] carbamoylamino] phenoxy]-N-methyl-pyridine-2-carboxamide.
